# Novel Strategies in C-H Oxidations for Natural Product Diversification—A Remote Functionalization Application Summary

**DOI:** 10.3389/fchem.2021.737530

**Published:** 2021-10-05

**Authors:** Huang Junrong, Yang Min, Dai Chuan, Zhou Yajun, Fang Huilong, Zhu Lizhi, Yin Feng, Li Zigang

**Affiliations:** ^1^ Department of Pharmacy, Shenzhen Second People’s Hospital (Shenzhen Institute of Translational Medicine), The First Affiliated Hospital of Shenzhen University, Shenzhen, China; ^2^ Shenzhen Bay Laboratory, Pingshan Translational Medicine Center, Shenzhen, China; ^3^ Department of Pathogenic Biology and Immunology, Xiangnan University, Chenzhou, China; ^4^ State Key Laboratory of Chemical Oncogenomics, School of Chemical Biology and Biotechnology, Peking University Shenzhen Graduate School, Shenzhen, China

**Keywords:** remote functionalization, C-H oxidation, natural product diversification, medicinal chemistry, total synthesis

## Abstract

Selectively activating the distal inactive C-H bond for functionalization is one of the on-going challenge in organic synthetic chemistry. In recent years, benefiting from the development of selective synthesis methods, novel methodologies not only make it possible to break non-traditional chemical bonds and attain more diversity in inactive sites, but also provide more possibilities for the diversification of complex natural products. Direct C-H bond functionalization approaches make it feasible to explore structure-activity relationship (SAR), generate metabolites and derivatives, and prepare biological probes. Among them, direct oxidation of inert C-H bonds is one of the most common methods for natural product diversification. In this review, we focus on the application of remote functionalization of inert C-H bonds for natural products derivatization, including the establishment of oxidation methods, the regulation of reaction sites, and the biological activities of derivatives. We highlight the challenges and opportunities of remote functionalization of inert C-H bonds for natural product diversification through selected and representative examples. We try to show that inert C-H bond oxidation, properly regulated and optimized, can be a powerful and efficient strategy in both synthetic and medicinal chemistry.

## Introduction

The evaluation of pharmacological activity and druggability are two main elements in developing new drugs. (Hopkins et al., 2008) The druggability of drug molecules is supported by their physicochemical, biochemical, pharmacokinetic, and safety properties. (Drews et al., 2000) New drug research with natural medicinal chemistry as a precursor is one of the key routes to drug development. (Harvey et al., 2008) Natural active substances often have good biological activity but do not meet the requirements for druggability properties and require further structural modification and optimization. (Bauer et al., 2014; Xiao et al., 2016; Blakemore et al., 2018) Due to the diversity of functional group substitution and the complexity of the molecular structure of natural drugs, it is long and challenging to achieve their total synthesis, which is not conducive to the rapid realization of drug molecule conformation analysis, mechanism of action, and target identification, and optimization of the physical and chemical properties before drug discovery. (Dias et al., 2012; Yao et al., 2017) Therefore, the use of late derivatization of natural products with selective functional group modification of different sites to achieve the diversity-directed synthesis of drug molecules has become an essential strategy in current research. (Durak et al., 2016)

For many years, chemists have been searching for new methodologies that can use natural products’ late derivatization to achieve efficient functionalization selectively. (Börgel et al., 2020; Gandeepan et al., 2019) A variety of C-H bonds are commonly found in natural products, and the physical and chemical properties of the C-H bonds in the non-activated state differ little from each other (Gutekunst and Baran, 2011). Achieving remote functionalization of non-activated C-H bonds of natural drug molecules has become a driving force for organic chemists to develop new methodologies. (Cernak et al., 2016) Currently, chemists have cleverly exploited the differences in electronic, site resistance, and stereoelectronic effects between C-H bonds in complex molecular environments to design catalysts/enzymes/auxiliaries for selective oxidation to construct C-O bonds offering new perspectives for realization in the late derivatization of natural products.

## C-H Oxidations for Natural Product Diversification

**O-insertion Reactions:** The use of stoichiometric reagents (hydroxyl/alkoxy radicals and organic oxidants) for C-H hydroxylation is a traditional method with a long history. (White et al., 2012) Stoichiometric electrophilic organic heterocycles (e.g., dioxiranes, oxazidine) are weak oxidizing agents mainly used in the hydroxylation of tertiary C-H. Although stoichiometric reactions with organic oxidants were limited by preparation challenges to adjust oxidant reactivity and selectivity at an early stage, the field has undergone a dramatic change, moving into modifications of natural products in recent years. (Fusco et al., 2006) Dioxirane today serve as one of the most powerful tools in organic chemistry, with numerous applications in commercially processes. They are quickly recognized as efficient oxygen transfer agents, especially for epoxidations and for a wide range of O-insertion reactions into C-H bonds. (Fusco et al., 2020)

**FIGURE 1:**
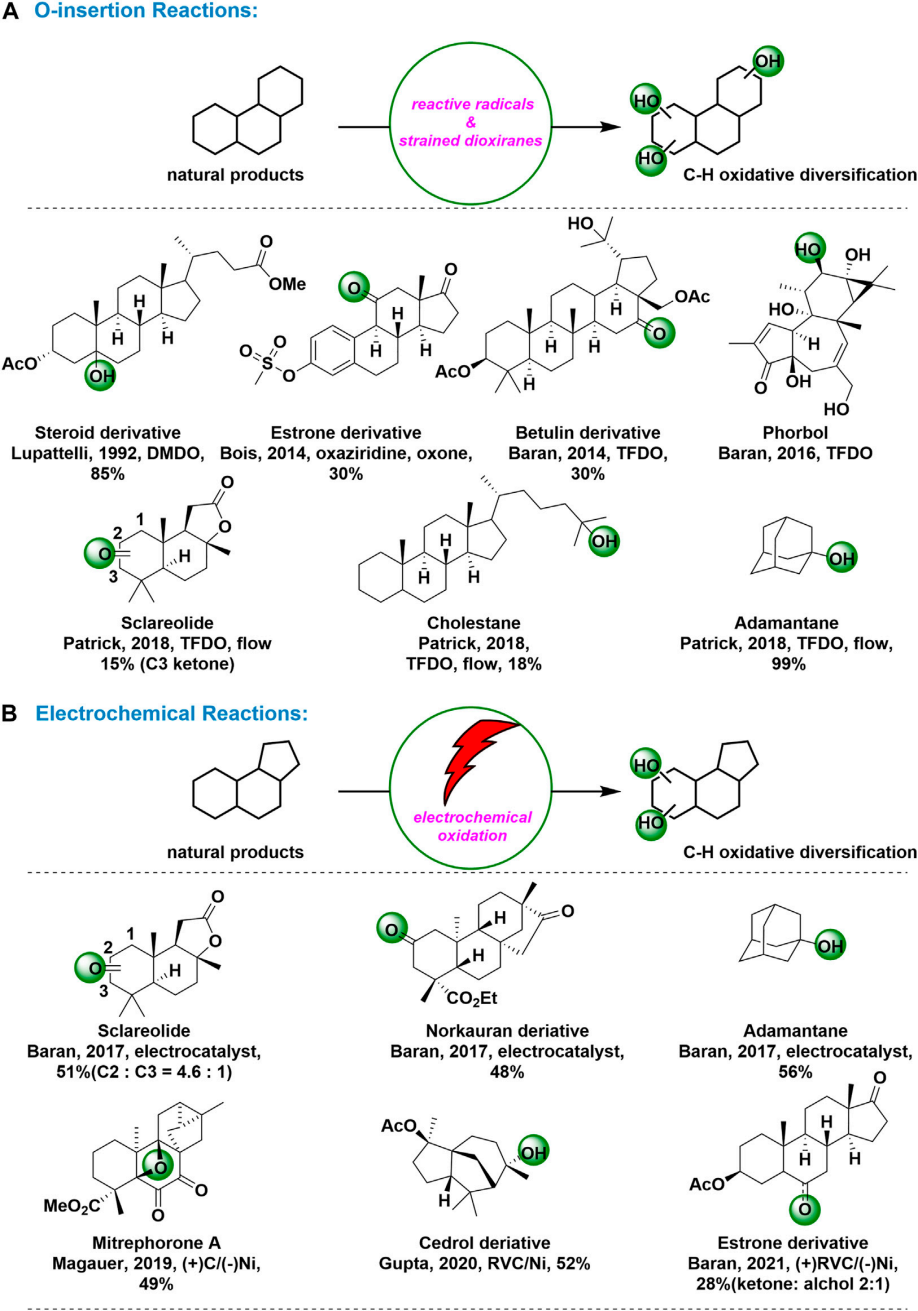
C-H Oxidations for natural products and complex molecules: **(A)** O-insertion reactions for C-H oxidation; **(B)** Electrochemical reactions for C-H oxidation.

As early as in 1992, Lupattelli group reported that the steroselective introduction of the hydroxyl moiety on C5 steroidic carbon was achieved by using DMDO as oxyfunctionalixing reagent. (Lupattelli et al., 1992) The C5 oxygen functionalization on methylprostatanes by dioxanes was apparently due to the favorable steric environment at this position. In 2014, Bois and coworkers reported a selective hydroxylation of tertiary and benzylic C–H bonds using a non-metal-based catalyst system, Oxone as the terminal oxidant, and an aqueous fluoroalcohol solvent mixture. (Adams and Bois, 2014) With the optimized condition, the C11-ketone derivative of estrone formed through the C9-alcohol, a product demonstrated to be prone to C9–C11 alkene formation. Epoxidation of the putative alkene under the reaction conditions and subsequent 1,2-shift would yield the C11-ketone derivative Figure 1A.

In 2014, Baran’s group attained the C16 ketone analog of botulin in 30% yield as a major product by using TFDO. No minor products were isolated in quantities sufficient for characterization, but none of these products contained a ketone motif. (Baran et al., 2014) Based on ^13^C NMR and computational chemical evaluation of electron rich methylene at C6 to C16, C16-H is found to be 3.5 kcal mol-1 lower than that of C6-H and is considerably lower than activation energies computed for all other positions. Although TFDO has previously been used for selective oxidation of sesquiterpene skeletons in total synthesis, this reaction constituted the first example of selective oxidation of methylene in a complex triterpene substrate. In 2016, Baran and coworkers reported a fabulous O-insertion reactions at C12 in their excellent total synthesis of (+)-phorbol, which required the selective oxidation of a methylene position in the presence of an enone, six tertiary C–H bonds and two other competing methylene sites. (Baran et al., 2016) To select the right oxidant for this transformation, they performed a computational analysis of its structure and inferred its inherent reactivity from NMR, predicting that the pseudo-equatorial C-H bond at C12 was the most active. Figure 1A. Finally, the small, reactive, electrophilic oxidant TFDO were selected, owing to its straightforward preparation and success in other challenging methylene oxidations. As predicted, TFDO cleanly achieved C–H oxidation at C12 to deliver the intermediate cyclopropyl carbinol intermediate. In 2018, Patrick and coworkers reported a new general TFDO-mediated Csp^3^-H oxidation method has been enabled in a fast, efficient and simple fashion through a continuous flow process. (Patrick et al., 2018) Figure 1A. By using the *in situ* generation of TFDO gas in continuous flow platform, the Csp^3^-H oxidation was achieved exclusively at the more sterically hindered Csp^3^-H site in moderate to excellent yields in a panel of adamantane derivatives. Furthermore, in similar conditions, the Csp^3^-H oxidation was selectively observed at the secondary carbon position C3 whereas for cholestane the transformation occurred preferentially at the terminal tertiary position Figure 1A.

**Electrochemical Reactions:** Over the past century, the attraction and promise of synthetic organic electrochemistry has been highlighted. In REDOX chemistry, which is often encountered in the formation of new bonds, it is difficult to think of a more economical way to add or remove electrons than electrochemistry. (Baran et al., 2019) Taking advantage of the unique reactivity advantage of this ancient REDOX regulation technology, it will be possible to make new chemical bonds with a higher level of chemical and regional selectivity. Indeed, some practical electrochemical oxidations of unactivated C−H bonds have been developed and applied to derivation of complex molecules. (Ackermann et al., 2021)

In 2017, Baran and coworkers reported a new class of reactive radicals with tunable reactivity and selectivity for unactivated C−H oxidation. (Baran et al., 2017) Based on a first-principles approach guided by computation, the new mediums were identified and rapidly expanded into a library using common building blocks and trivial compositing techniques. This practical method used quinolidine as a medium in a simple electrochemical setup using inexpensive carbon and nickel electrodes. A wide variety of functional groups have been shown to be compatible with chemical selection processes. The scalability of this approach, demonstrated by the successful oxidation of sclareolide at a scale of 50 g, has the potential to facilitate the synthesis of complex materials. In addition, this quinuclidine-mediated protocol could also be used to oxidize tertiary C−H bonds, like adamantine, affording the corresponding alcohols as products Figure 1B.

In 2019, the Magauer group developed a synthetic route to (−)-mitrephorone A via a bioinspired late stage C−H oxidation of (−)-mitrephorone B. (Magauer et al., 2019) Figure 1B. According to report, exposure of mitrephorone B to electrochemical oxidative condition described by Baran, mitrephorone A could be attained in 49% yield, which was considered as the first example of oxetane formation via C−H oxidation. In 2020, Gupta and coworkers reported an efficient electrochemical method for the selective oxidation of C–H bonds of unactivated alkanes. (Gupta et al., 2020) Figure 1B. using a biomimetic iron complex, as the redox mediator in an undivided electrochemical cell with inexpensive carbon and nickel electrodes, a natural product derivative of cedrol, having a rigid structure with tertiary C-H bonds, affords a single hydroxylated product in 52% yield. Recently, Baran group reported a new class of reactive radicals with tunable reactivity and selectivity for unactivated C−H oxidation. (Baran et al., 2021) Figure 1B. N-ammonium ylides-mediated electrochemical C-H oxidation worked with a wide range of substrates and revealed their unusual and often unique selective properties. The ability of ylide mediators to oxidize unactivated methylene positions in estrone derivatives was remarkable, where other commonly employed chemical reagents failed.

**Organometallic Reactions and Cytochrome P-450 Mimics:** Distinct from those of the free radicals or dioxiranes, metal complex-mediated C-H bond oxidation show remarkable site selectivity and favor oxidation at very strong primary C-H bonds. (White et al., 2018) The selective, nondirectional oxidation of inactive hydrocarbons by cytochrome P-450 enzymes, like Iron and Manganese porphyrins, have stimulated interest in the development of chemical model systems. In addition, metal-mediated non-activated C-H bond functionalization has developed dramatically and enabled the emerging area of late-stage C-H functionalizations for synthesis and derivatizing complex molecules. (Lei et al., 2019)

**FIGURE 2:**
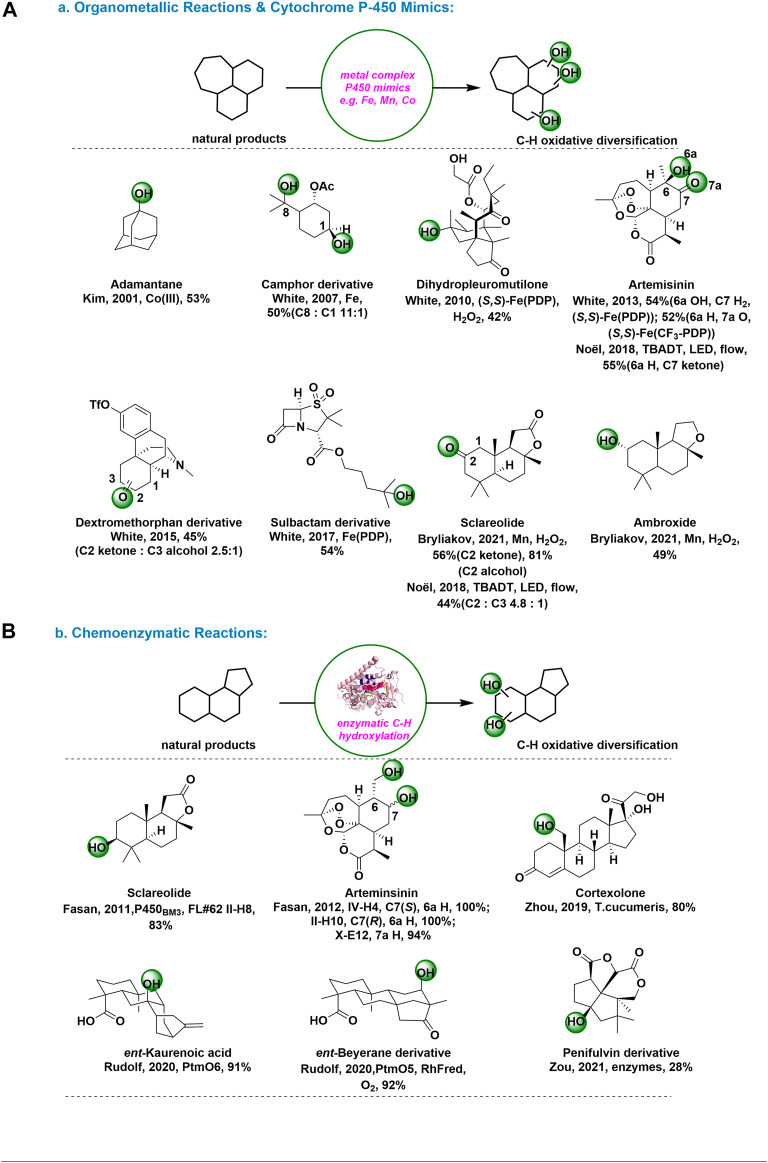
C-H Oxidations for natural products and complex molecules: **(A)** organometallic reactions and cytochrome P-450 mimics; **(B)** Chemoenzymatic reactions.

In 2001, Kim and coworkers disclosed an regioselective alkane hydroxylation approach by using Co(TPFPP) (CF_3_SO_3_) [TPFPP = meso-tetrakis (pentafluorophenyl) porphinato dianion] and *m*-CPBA, in which a high degree of selectivity for tertiary C-H bond over secondary C–H bond was observed in the hydroxylation of adamantine. (Kim et al., 2001) Figure 2A. In the past decade or so, White and coworkers have developed a series of non-heme iron hydroxylation catalysts, like Fe(PDP) and Fe(CF_3_-PDP), for C-H bond oxidation with high site-selectivity and chemoselectivity. The development of quantitative structure-based catalyst reactivity models led to more targeted application of C-H oxidations at late stages of complex molecule synthesis and enabled site-divergent diversification of bioactive molecules. With the optimized, (–)-acetoxy-*p*-menthane was oxidized (C8:C1 = 11:1), which demonstrate that in present of C–H bonds with similar electron densities, sterics can provide a second handle for selectivity. (White et al., 2007) These results suggest that steric hindrance can provide an alternative treatment for selectivity in molecules with carbon-hydrogen bonds of similar electron density. Figure 2A. Furthermore, with the well tune iron catalyst system, White’s group achieved methylene C–H bond oxidations in diverse natural-product settings with predictable and high chemo-, site-, and even diastereoselectivities. Some outstanding achievements, including oxidation of (−)-dihydropleuromutilone with Fe(S,S-PDP) (White et al., 2010), oxidation of (+)-artemisinin with (*R*,*R*)-Fe(PDP) and (*R*,*R*)-Fe(CF3-PDP) to different derivatives (White et al., 2013), and remote late-stage oxidation of dextromethorphan derivative (White et al., 2015) and sulbactam derivative (White et al., 2017), have been widely recognized Figure 2A.

On the other hand, Noël and coworkers developed a mild and selective Csp^3^-H aerobic oxidation enabled by decatungstate photocatalysis in 2018. (Noël et al., 2018) This steric hindation-driven selectivity is further demonstrated in the oxidation of Sclareolide, where oxidation preferentially occurs at the C2 position. Figure 2A. The ability to selectively oxidize natural scaffolds, such as (+)-sclareolide, and artemisinin, illustrated the practicability of the new method. Recently, a late-stage C-H oxofunctionalization of (-)-ambroxide in synthetically useful yield with H_2_O_2_ in the presence of bioinspired nonheme Mn complexes was reported by Bryliakov’s group. (Bryliakov et al., 2021) Figure 2A.

**Chemoenzymatic Reactions:** In recent years, efforts to access natural products and their derivatives have begun to explore the possibility of combining organic synthesis tools with biosynthesiology for efficient chemoenzymatic synthesis. (Renata et al., 2017) This approach has the unique advantage of being able to solve challenging chemical and stereoselective problems in organic chemistry by discovering new enzymes with unique reactivity and functional group compatibility, while at the same time relying on advances in modern chemical synthesis for efficient preparation of blocks or further downstream operations. (Arnold et al., 2011) Among them, chemoenzymatic platforms to access highly oxidized natural product by strategically enzymatic oxidation methods have been disclosed and gained considerable development. (Renata et al., 2019)

In 2011 and 2012, the Fasan group used various P450 variants for selective hydroxylation of sclareolide and artemisinin. (Fasan et al., 2011-2012) Figure 2B. This method could provide an efficient solution to the problem of developing P450 catalysts with fine-tuned site-selectivity for the oxidative activation of multiple, isolated sp^3^ C−H bonds in a complex molecule. In 2019, Zhou group reported a highly efficient method to synthesize 19-OH-cortexolone in 80% efficiency at the multi-gram scale using *T*. cucumeris. (Zhou et al., 2019) Figure 2B. Using this biocatalytic C19-hydroxylation method, the unified synthesis of six C19-hydroxylated pregnanes was achieved in just 4 to 9 steps.

In 2020, Rudolf group reported using PtmO5-RhFRed on ent-beyerane at C11 and PtmO6 on ent-kaurenoic acid at C7 hydroxylation, delivering the product as a single diastereomer with good conversion and yield. (Rudolf et al., 2020) Figure 2B. Using this chemoenzymatic strategy can minimal functional group interconversions and protecting group manipulations. Recently, Zou group identified two parallel and complex pathways (peni and aspe) of fungal dioxafenestrane sesquiterpenes from terrestrial fungus and marine-derived fungus, respectively. (Zou et al., 2021) Figure 2B. These two pathways shared a common intermediate but were different in late-stage tailoring steps such as Baeyer-Villiger oxidation in δ-lactone formation and hydroxylation carried on non-activated carbons. This method especially provided diverse strategies and valuable biocatalysts for nonactivated carbon oxidation modification.

## Summary and Outlook

Remote non-activated C-H bond selective functionalization methodologies can provide more options for implementing late-stage derivatization of natural products, thus greatly facilitating the development process of natural drugs towards clinical applications. This review provides a detailed overview of remote inert C-H oxidation applications for natural product diversification, summarizes the types and patterns of reactions, and provides a systematic approach for future exploration of new methodologies.

Although very significant progress has been made in the study of hydrocarbon bond activation, it appears from the above presentation that most of the reactions have been limited to a few specific systems and are still far from universal hydrocarbon bond activation in a general sense, especially an available method that can be used universally for the late functionalization of natural drugs. Hydrocarbon bond activation, the holy grail of chemical reactions, will continue to receive a great deal of attention from chemists.
